# A Clinical Trial about a Food Supplement Containing α-Lipoic Acid on Oxidative Stress Markers in Type 2 Diabetic Patients

**DOI:** 10.3390/ijms17111802

**Published:** 2016-10-28

**Authors:** Giuseppe Derosa, Angela D’Angelo, Davide Romano, Pamela Maffioli

**Affiliations:** 1 Centre of Diabetes and Metabolic Diseases, Department of Internal Medicine and Therapeutics, University of Pavia and Fondazione IRCCS Policlinico S. Matteo, 2-27100 Pavia, Italy; labmedmol@smatteo.pv.it (A.D.); dr.davideromano85@gmail.com (D.R.); pamelamaffioli@hotmail.it (P.M.); 2 Center for Prevention, Surveillance, Diagnosis and Treatment of Rare Diseases, Fondazione IRCCS Policlinico S. Matteo, 2-27100 Pavia, Italy; 3 Center for the Study of Endocrine-Metabolic Pathophysiology and Clinical Research, University of Pavia, 2-27100 Pavia, Italy; 4 Laboratory of Molecular Medicine, University of Pavia, 2-27100 Pavia, Italy; 5 PhD School in Experimental Medicine, University of Pavia, 2-27100 Pavia, Italy

**Keywords:** α-lipoic acid, *L*-carnosin, malondialdehyde, oxidative stress

## Abstract

The aim of this study was to evaluate the effect of a food supplement containing α-lipoic acid and of a placebo on glyco-metabolic control and on oxidative stress markers in type 2 diabetics. We randomized 105 diabetics to either a supplementation containing 600 mg of α-lipoic acid, 165 mg of *L*-carnosin, 7.5 mg of zinc, and vitamins of group B, or a placebo, for three months. We evaluated body mass index, fasting plasma glucose (FPG), post-prandial-glucose (PPG), glycated hemoglobin (HbA_1c_), fasting plasma insulin (FPI), HOMA-index (HOMA-IR), lipid profile, high sensitivity C-reactive protein (Hs-CRP), superoxide dismutase (SOD), glutathione peroxidase (GSH-Px), malondialdehyde (MDA). There was a reduction of FPG, PPG, and HbA_1c_ with the food supplement containing α-lipoic acid compared with a baseline, and with the placebo. Concerning lipid profile, we observed a reduction of LDL-C, and Tg with the food supplement, compared with both the baseline, and the placebo. There was a reduction of Hs-CRP with the food supplement containing α-lipoic acid, both compared with the baseline and the placebo. An increase of SOD, and GSH-Px, and a decrease of MDA were reached by the food supplement containing α-lipoic acid, both compared with the baseline and the placebo. We can conclude that the food supplement containing α-lipoic acid, *L*-carnosin, zinc, and vitamins of group B improved glycemic control, lipid profile, and anti-oxidative stress markers.

## 1. Introduction

**Figure 1:**
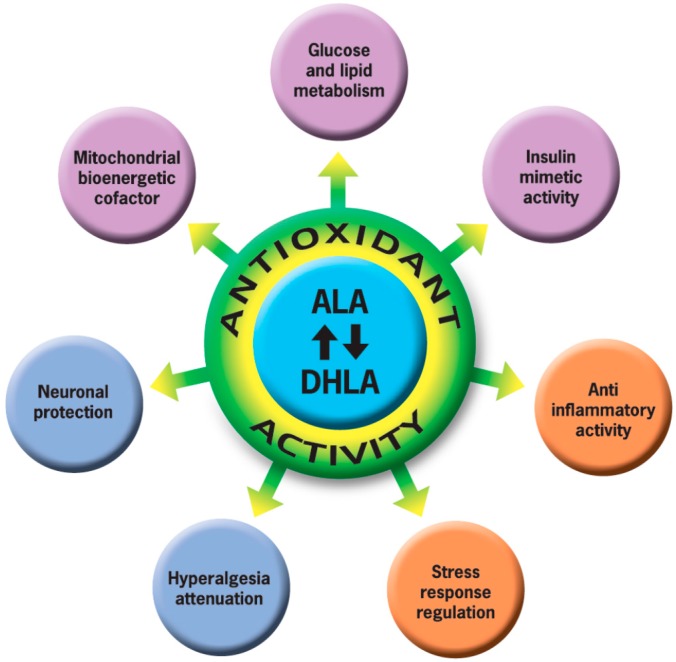
Biological functions of α-lipoic acid. α-lipoic acid (ALA) and its reduced form dihydrolipoic acid (DHLA) create a potent redox couple, often called a “universal anti-oxidant” for its capacity to regenerate several others anti-oxidants, such as vitamins C and E and glutathione. α-lipoic acid is also able to directly scavenge ROS, possesses metal chelating activity, and enhances the mitochondrial expression of key anti-oxidant enzymes. Through these properties, α-lipoic acid exerts different activities, from mitochondrial bioenergetics cofactor to stress response regulation and neuronal protection. Mitochondrial bioenergetic cofactor: α-lipoic acid is a key cofactor for mitochondrial bioenergetic enzymes, including pyruvate dehydrogenase and α-ketoglutarate dehydrogenase complexes, stimulating glucose and lipid metabolism. It acts as an insulin-mimetic agent, regulating the IR/PI3K/Akt pathway, so it enhances the uptake and the utilization of glucose, improving glycemic control. Stress response regulation: α-lipoic acid responds to stress factors, inhibiting stress induced transcription factor activation, such as NF-κB and AP-1, and modulating pro-inflammatory signaling, hence the anti-inflammatory activity. Neuronal protection: it is also well known that α-lipoic acid improves diabetic polyneuropathies, while attention has only recently been focused on the capacity to attenuate the hyperalgesia through the modulation of T-type calcium and transient receptor potential (TRPA1) channels.

Type 2 diabetes mellitus is considered a risk factor for cardiovascular disease. Over 50% of diabetic patients have clinical manifestations of diabetic neuropathy. Diabetic neuropathy is defined by a progressive loss of nerve fibers due to a condition of chronic hyperglycemia; genetic and environmental factors also play an important role. Among all diabetic neuropathy cases, about 80% seems to be distal, symmetrical sensitive-motor neuropathy [1,2]. α-lipoic acid is the treatment of choice for treating diabetic neuropathy, in combination with symptomatic therapy and physical treatment. α-lipoic acid plays the main role as the anti-oxidant (Figure 1) and metabolic component of some enzymatic reactions involved in glucose metabolism [3]. Endogenously synthesized α-lipoic acid is covalently bound to specific proteins, cofactors for mitochondrial dehydrogenase enzyme complexes [4]. In addition, there is an increasing interest in potential therapeutic uses of pharmacological doses of free α-lipoic acid [5]. α-lipoic acid’s anti-oxidant properties are due to several factors, including (1) its capacity to scavenge reactive oxygen species (ROS) directly; (2) its ability to regenerate endogenous anti-oxidants, such as glutathione and vitamins E and C; and (3) its metal-chelating activity, resulting in reduced ROS production. Moreover, due to its anti-oxidant properties, α-lipoic acid has recently been reported to afford protection against oxidative injury in various disease processes, including neurodegenerative disorders [6]. The efficacy of α-lipoic acid was confirmed by the SIDNEY 2 study [7], where results showed that oral treatment with α-lipoic acid taken for five weeks resulted in an improvement of neuropathic symptoms in patients affected by distal symmetric polyneuropathy. Regarding the mechanism through which neuropathy develops, previously published papers showed that hyperglycemia generates free radicals, contributing to the development and progression of diabetes complications. There is large evidence that there is a relationship between the severity of diabetic neuropathy and the frequency and duration of hyperglycemic periods [8]. For this reason, improving oxidative stress may be a way to reduce diabetic complications [9,10], and this is probably the way α-lipoic acid acts. However, studies aimed to confirm the effects of α-lipoic acid on glycemic control and on anti-oxidant parameters in humans are lacking.

For this reason, the aim of this study was to evaluate the effect of food supplements containing 600 mg of α-lipoic acid, 165 mg of *L*-carnosin, 7.5 mg of zinc, and vitamins of group B, and that of a placebo, taken once a day for three months, on glyco-metabolic control and on oxidative markers in type 2 diabetic patients.

## 2. Results

### 2.1. Study Sample

We enrolled 105 patients in this trial, 54 of which were randomized to the food supplement containing alpha-lipoic and 51 of which were randomized to the placebo. One patient in the group treated with food supplement, and two patients treated with the placebo did not complete the study; the reason for the premature withdrawal was lost to follow-up for all the three patients. Study population characteristics are described in Table 1.

#### 2.1.1. Body Weight

We did not record any variations in body weight or BMI.

#### 2.1.2. Glycemic Control

There was a reduction of FPG, PPG, and HbA_1c_ with the food supplement containing α-lipoic acid compared with the baseline (*p* < 0.05 for all) and with the placebo (*p* < 0.05 for all). There was a reduction in HOMA-IR, compared with both the baseline and the placebo (*p* < 0.05 for both).

### 2.2. Lipid Profile

We recorded a decrease in LDL-C and TG with the food supplement containing α-lipoic acid, compared with both the baseline (*p* < 0.05) and the placebo (*p* < 0.05 for both).

### 2.3. Inflammatory and Oxidative Parameters

A reduction of Hs-CRP was reduced by the food supplement containing α-lipoic acid, both compared with the baseline and with the placebo (*p* < 0.05 for both). An increase of SOD, and GSH-Px, and a decrease of MDA were reached with the food supplement containing α-lipoic acid, compared with both the baseline and the placebo.

### 2.4. Side Effects

No side effects were reported. No patients interrupted the food supplement containing α-lipoic acid due to side effects.

## 3. Discussion

Diabetes and insulin resistance are involved in the onset of cardiovascular and nervous diseases. The mechanism responsible for these disorders involves, mainly, oxidative stress generated by reactive oxygen species (ROS) and reactive nitrogen species (RNS). It has been largely reported that diabetes impairs endothelial nitric oxide synthase (eNOS) activity, thus increasing ROS production, with a consequent reduction of NO bioavailability. α-lipoic acid has shown beneficial effects both in the prevention and in the treatment of diabetes because of its insulin-mimetic and anti-inflammatory action; it is also involved in mitochondrial bioenergetic reactions [11]. The positive effects of α-lipoic acid on oxidative stress parameters have been shown by our study: α-lipoic acid increased SOD, an enzyme with the role of commuting highly reactive O^2−^ into H_2_O_2_, which is subsequently reduced in H_2_O by mitochondrial glutathione peroxidase and catalase [12]. Throughout this mechanism, SOD neutralizes superoxide radicals, as reported in diabetic peripheral nerve tissue [13,14,15]. Furthermore, SOD inhibits inflammatory response, with a consequent reduction of hyperalgesia, typical of diabetic neuropathy [16]. In our study, α-lipoic acid also reduced GSH-Px, whose main biological role is to protect the organism from oxidative damage.

In our study, we recorded an improvement of glycemic control and insulin resistance with α-lipoic acid supplementation. This effect of α-lipoic acid on glycemic control is in line with reported by Ansar et al. [17], which similarly reported an improvement of FPG, PPG, and HOMA-index. These effects may be due to the insulin signaling pathway, with an increase in PI 3-kinase and protein kinase B (Akt) [18,19,20]. The α-lipoic acid increased intrinsic activity of GLUT in an insulin-like manner, even if p38 mitogen-activated protein kinase may also mediate the activation of GLUT. Chronic α-lipoic acid treatment improved both insulin stimulated glucose oxidation and glycogen synthesis; consequently, it is related to decreased levels of insulin and free fatty acids.

Concerning the effects of α-lipoic acid on LDL-C and Tg levels, they have been previously reported as acting on rats by Thirunavukkarasu et al. [21]. These results might be due to the Alfa lipoic acid effect on glucose utilization reported above and to the consequent reduced levels of free fatty acids.

## 4. Material and Methods

### 4.1. Study Design

This randomized, double-blind, placebo-controlled trial took place at the Centre of Diabetes and Metabolic Diseases, Department of Internal Medicine and Therapeutics, University of Pavia and Fondazione IRCCS Policlinico S. Matteo, Pavia, Italy.

The study protocol was conducted in accordance with the Declaration of Helsinki and its amendments, and was approved by the local review board. Suitable patients were identified from case notes, computerized clinic registers, or both and were contacted by the clinicians personally or by telephone. All eligible candidates had to provide signed informed consent before enrollment in the study.

### 4.2. Patients

We enrolled 105 Caucasian adults, of both sexes, 18–75 years of age, with type 2 diabetes according to the ESC (European Society of Cardiology) and EASD (European Association for the Study of Diabetes) Guidelines criteria [22], and with a glycated hemoglobin (HbA_1c_) level >7.0%. All participants were overweight (BMI ≥25, and <30 kg/m^2^).

We excluded patients with previous ketoacidosis or unstable or rapidly progressive diabetic retinopathy, nephropathy, neuropathy, impaired hepatic or renal function, or severe anemia. Patients with a myocardial infarction or stroke within 6 months before study enrollment were not enrolled. Women with childbearing potential and not taking adequate contraceptive precautions were not considered for inclusion in this trial.

### 4.3. Treatments

Subjects fulfilling all inclusion criteria reported above were randomized to receive either, in a 1:1 ratio, a supplementation containing LICA^®^, a patented association of components (600 mg of α-lipoic acid, 165 mg of *L*-carnosin, and 7.5 mg of zinc) and vitamins of group B (Table 2) produced by DIFASS Italia (Prato, Italy), or a placebo, once a day for three months, in a single oral dose, with a glass of water.

The food supplement containing α-lipoic acid was added to the previously taken anti-diabetic therapy, including insulin. The methods used to ensure the blind status of the trial, to perform randomization and to assess medical compliance, have been previously described [23]. Patients did not sustain any costs for the medications provided.

### 4.4. Diet and Exercise

Subjects followed a controlled-energy diet according to the American Heart Association (AHA) recommendations [24].

Standard diet advice was given by a dietician, a specialist doctor, or both as previously described [23].

### 4.5. Assessments

At the start of the study, patients were interviewed about their medical history, underwent physical examination, examined for vital signs, and underwent a 12-lead electrocardiogram. We evaluated body mass index (BMI), fasting plasma glucose (FPG), post-prandial-glucose (PPG), glycated hemoglobin (HbA_1c_), fasting plasma insulin (FPI), HOMA-index (HOMA-IR), total cholesterol (TC), low-density lipoprotein cholesterol (LDL-C), high-density lipoprotein cholesterol (HDL-C), triglycerides (TG), high sensitivity C-reactive protein (Hs-CRP), superoxide dismutase (SOD), glutathione peroxidase (GSH-Px), and malondialdehyde (MDA).

We measured these parameters both at the baseline and after 3 months of the food supplement containing α-lipoic acid or the placebo. All adverse events were recorded to assess tolerability. For a complete description of how various parameters were assessed, see our previous papers [23,25]. Superoxide dismutase (SOD) was evaluated according to the method described by Marklund et al. [26]. We assessed GSH-Px activity according to Paglia et al. [27]. Malondialdehyde was obtained via acid hydrolysis of 1,1,3,3-tetraethoxy-propane according to Requena et al. [28].

### 4.6. Statistical Analysis

We conducted an intention-to-treat (ITT) analysis in patients receiving ≥1 dose of medication. The statistical significance of the independent effects of treatments on each variable was determined using ANCOVA. We used a 1-sample *t*-test to compare values obtained before and after treatment, and 2-sample *t*-tests for between-group comparisons. Statistical analysis of data was performed using the Statistical Package for Social Sciences software version 11.0 (SPSS Inc., Chicago, IL, USA). Data in the text were presented as mean (±standard deviation). A *p*-value of <0.05 was considered statistically significant [29].

## 5. Conclusions

The use of a food supplement containing α-lipoic acid seems to bring an improvement in glycemic control and in lipid profile, even if only slightly. Moreover, α-lipoic acid seems to improve markers of oxidative stress, such as GSH-Px, SOD, and MDA, and of inflammation (Hs-CRP).

## Figures and Tables

**Table 1:** Mean changes during the study.

Parameters	Food Supplement Containing Alpha-Lipoic Acid		Placebo	
–	Baseline	3 Months	Baseline	3 Months
Number	54	53	51	49
Sex (Male/Female)	26/28	25/28	25/26	24/25
Age (years)	52.5 ± 7.9	–	53.1 ± 8.2	–
Smoking status (M/F)	12/10	11/10	13/12	13/12
Height (m)	1.68 ± 0.05	–	1.69 ± 0.06	–
Weight (kg)	80.1 ± 7.1	79.8 ± 6.9	80.4 ± 7.3	80.9 ± 7.6
BMI (kg/m^2^)	28.4 ± 2.5	28.3 ± 2.4	28.1 ± 2.2	28.3 ± 2.4
FPG (mg/dL)	119.5 ± 16.3	102.2 ± 9.6 *^,^°	122.3 ± 17.1	120.4 ± 16.6
PPG (mg/dL)	164.2 ± 22.1	141.3 ± 18.4 *^,^°	162.3 ± 21.2	159.1 ± 19.8
HbA_1c_ (%)	7.8 ± 0.4	7.2 ± 0.3 *^,^°	7.9 ± 0.5	7.7 ± 0.3
FPI (µU/mL)	11.1 ± 3.6	10.6 ± 3.3	10.2 ± 2.6	10.4 ± 2.8
HOMA-IR	3.26 ± 1.86	2.67 ± 1.23 *^,^°	3.08 ± 1.64	3.09 ± 1.71
TC (mg/dL)	192.5 ± 17.3	171.3 ± 14.6 *^,^°	191.7 ± 16.8	193.4 ± 17.7
LDL-C (mg/dL)	123.2 ± 11.5	105.7 ± 9.8 *^,^°	122.5 ± 11.0	124.9 ± 11.8
HDL-C (mg/dL)	45.7 ± 5.5	46.1 ± 5.7	45.9 ± 5.6	45.6 ± 5.4
Tg (mg/dL)	118.2 ± 25.8	97.4 ± 19.5 *^,^°	116.5 ± 24.2	114.7 ± 22.2
Hs-CRP (mg/L)	2.3 ± 0.6	1.8 ± 0.3 *^,^°	2.5 ± 0.8	2.2 ± 0.7
SOD (U/mL)	94.7 ± 19.3	111.4 ± 24.9 *^,^°	96.2 ± 21.4	98.1 ± 22.3
GSH-Px (EE/U)	97.2 ± 39.8	119.6 ± 43.3 *^,^°	93.7 ± 36.5	94.4 ± 35.1
MDA (nmol/mL)	42.8 ± 18.5	33.9 ± 14.8 *^,^°	44.6 ± 22.7	41.5 ± 20.8

Data are expressed as mean ± standard deviation; * *p* < 0.05 vs. baseline; ° *p* < 0.05 vs. placebo. BMI: body mass index; FPG: fasting plasma glucose; PPG: post-prandial-glucose; HbA_1c_: glycated hemoglobin; FPI: fasting plasma insulin; HOMA-IR: HOMA-index; TC: total cholesterol; LDL-C: low-density lipoprotein cholesterol; HDL-C: high-density lipoprotein cholesterol; Tg: triglycerides; Hs-CRP: high sensitivity C-reactive protein; SOD: superoxide dismutase; GSH-Px: glutathione peroxidase; MDA: malondialdehyde.

**Table 2:** Food supplement composition.

Components	Dose
α-lipoic acid	600 mg
*L*-carnosine	165 mg
Zinc	7.5 mg
Vitamin PP	9.0 mg
Vitamin B5	3.0 mg
Vitamin B6	1.0 mg
Vitamin B1	0.7 mg
Vitamin B2	0.8 mg
Vitamin B12	0.5 mcg
Folic acid	100 mcg
